# Health measures of *Eeyouch* (Cree) who are eligible to participate in the on-the-land Income Security Program in *Eeyou Istchee* (northern Quebec, Canada)

**DOI:** 10.1186/s12889-021-10654-7

**Published:** 2021-03-31

**Authors:** Robert J. Moriarity, Aleksandra M. Zuk, Eric N. Liberda, Leonard J. S. Tsuji

**Affiliations:** 1 Department of Physical and Environmental Sciences, SW151 University of Toronto, Toronto, ON M1C 1A4 Canada; 2 School of Nursing, Queen’s University, Kingston, ON Canada; 3 School of Occupational and Public Health, Ryerson University, Toronto, ON Canada

**Keywords:** Income Security Program, Indigenous Health, Cree peoples, Traditional cultural activities, Physical activity, Traditional diet, on-the-land activities

## Abstract

**Background:**

Participation in on-the-land programs that encourage traditional cultural activities may improve health and well-being. The Income Security Program (ISP) − a financial incentive-based on-the-land program − for *Eeyouch* (Cree) hunters and trappers in *Eeyou Istchee* was created as a result of the 1975 James Bay and Northern Quebec Agreement to help mitigate the effects of hydroelectric development on the Cree people of northern Quebec, Canada. Beyond the ISP’s financial incentives, little is known about the health measures of those who are eligible to participate in the ISP (i.e. spent ≥120 days on-the-land during the previous year). Therefore, this paper’s objective was to assess the health measures of northern Quebec Cree, who were eligible for participation in the ISP.

**Methods:**

Using participant data (*n* = 545) compiled from the *Nituuchischaayihtitaau Aschii* Multi-Community Environment-and-Health Study, we assessed 13 different health measures in generalized linear models with the independent variable being the eligibility to participate in the ISP.

**Results:**

Participants in the present study who were eligible for the ISP had significantly higher levels of vigorous and moderate activity per week, and higher concentrations of omega-3 polyunsaturated fatty acids in the blood compared to those ineligible for the ISP (i.e. spent ≤119 days on-the-land during the previous year). Encouragingly, following model adjustment for age and sex, participants eligible for the ISP did not have higher blood concentrations of mercury than those who were not eligible for the ISP.

**Conclusions:**

Our results suggest that the participants eligible for participation in the ISP are likely to be healthier than those who are ineligible to participate − and are promising for on-the-land programs for Indigenous peoples beyond a financial incentive − with no apparent higher risk of increasing contaminant body burden through traditional on-the-land-activities (e.g. fish consumption).

**Supplementary Information:**

The online version contains supplementary material available at 10.1186/s12889-021-10654-7.

## Background

Participating in traditional cultural activities and being on-the-land are important aspects of the Indigenous way of life [114, 115]. Traditional cultural activities can include hunting, fishing, gathering, trapping, and orally passing down traditional knowledge to younger generations [63, 117]. Hunting, fishing, and gathering also help offset the higher cost of foods in Indigenous communities, especially northern communities in Canada [39, 81, 116]. Importantly, studies have also shown that being on-the-land, that is, participating in traditional activities, may improve health and well-being [1, 36, 45, 46, 58, 79]. For example, approximately 70% of Alaskan Native adults who engaged in at least one traditional cultural activity per year self-reported benefits to their well-being as a result [92, 93]. Additionally, Alaskan Native women used the phrase ‘keeping busy’ to describe their perceptions of health and well-being, in the context of being on-the-land – while eating traditional food and respecting Elders and nature [45]. Further, in a small-prospective-cohort study [97] of eastern James Bay Cree Peoples (Quebec, Canada) with type 2 diabetes – 25 participants spent 3 months on-the-land, while 26 controls remained in the community – on-the-land living had positive effects on various health measures (e.g. activity increased, weight decreased, blood glucose decreased). Thus, programs that encourage traditional cultural activities on-the-land may improve health and well-being. One such program is the Income Security Program (ISP), available to the First Nations Cree Peoples of the *Eeyou Istchee* region, northern Quebec, Canada.

The ISP for Cree hunters and trappers in *Eeyou Istchee* was established by the James Bay and Northern Quebec Agreement (JBNQA) nearly 46 years ago “to provide an income guarantee and benefits…for Cree people who wish to pursue harvesting activities as a way of life” (section 30.1.1, [48]). This on-the-land program was negotiated to help mitigate hydroelectric mega-projects’ effects on the Cree Peoples traditional homelands, especially on their way of life and connection to the land [49]. For eligible participation in the ISP – participation is optional – community members of *Eeyou Istchee* are required to spend at least 120 days on-the-land involved in traditional cultural activities, such as, fishing and wildlife harvesting [98, 112]. Cree Peoples who are eligible for participation in the ISP are paid quarterly following interviews with the Cree Hunters and Trappers Income Security Board representatives where participants report their on-the-land activity and their benefits are calculated [24]. According to Altman and Cochrane [3], participating in an income-incentive program to support on-the-land activities has three benefits: (1) participants are paid no matter what type of harvest they obtain for any reason outside of their control; (2) it promotes wildlife management in the community by participants who are also community members; and (3) the program provides incentives for custodial stewardship of individual community traditional lands. To date, the ISP is the longest-running on-the-land financial incentive program in the world. However, there is a paucity of studies, beyond the financial incentive, of health measures for these types of on-the-land programs. Consequently, an assessment of the potential benefits of participation in on-the-land-incentive programs, such as the ISP, which may promote health and well-being through traditional cultural activities, is warranted, particularly from a health measures perspective. Therefore, the purpose of the present study was to assess several health measures of Cree Peoples who were eligible for participation in the ISP and assess if their potential participation based on their eligibility could be beneficial, beyond a financial standpoint, compared to the Cree Peoples who were not eligible for participation in the ISP.

## Methods

### Study region

**Fig. 1:**
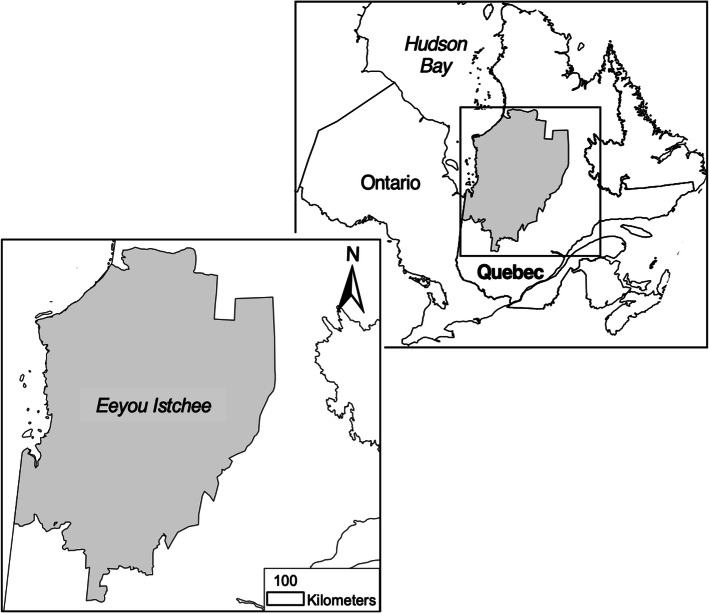
The *Eeyou Istchee* territory of Northern Quebec, Canada. The map depicted in Fig. 1 is wholly designed and owned by the corresponding author, Robert J. Moriarity

There are approximately 18,000 residents of *Eeyou Istchee* (Fig. 1) who speak at least one of the Cree, French or English languages [25, 106]. Traditional cultural activities in this region vary based on the community’s location as four communities are on the coast, and the other five are inland, where approximately 62% of the total population live in coastal communities and 48% live inland. The region is primarily accessible by road (paved and unpaved), air, and water, while the most northern coastal community of approximately 800 residents is only accessible by air or water.

### Data

The *Nituuchischaayihtitaau Aschii* Multi-Community Environment-and-Health Study in *Eeyou Istchee* [NA study] [75] was a seven-community study (exclusive of the two community pilot studies) carried out in the *Eeyou Istchee* region (Fig. 1) between 2005 and 2009 that investigated environmental health concerns of 1405 participants. Prior to the start of the multi-community study in 2005, “A Needs and Feasibility Study” was conducted with the remaining seven Cree Nations that would eventually take part in the multi-community study [76]. The main objectives of the Needs-and-Feasibility Study were to: “Carry out an intensive consultation process with all the Cree communities-and entities to determine local environment-and-health-issues…Develop a detailed study proposal that includes the information and direction received during the local consultations.” ([77], p. 2). From 2003 to 2004, consultations occurred with these regional Cree entities: the Cree Trappers’ Association Executive and Board; the James Bay Advisory Committee on the Environment; and the Traditional Pursuits Division of the Cree Regional Authority [77]. Similarly, consultative meetings were held with locally-elected Cree governing bodies – that is, Chiefs and Councils – as well as with community members in the Cree Nations themselves [77]. These consultations helped to inform the proposal as presented to the funding agency [77]. It should also be noted that Chiefs and Councils formally approved the project [75]. For more information on the multi-community study population, study design, and data collection, please refer to Bonnier-Viger et al. [13] and Nieboer et al. [74, 75]. Dr. Leonard Tsuji was involved at the community level during the pilot-study and has over 30 years of experience working with James Bay Cree communities.

Ethics approval was granted by McGill University, Laval University, McMaster University, and the Cree Board of Health and Social Services of James Bay (CBHSSJB). Participants provided written informed consent in Cree, English, or French languages. Additionally, the university researchers entered a “Research and Data Use Agreement” [21] whereby the CBHSSJB was “the guardian of the Original Data” (p. 7); and “the CBHSSJB takes decisions for the benefit of the Cree participants, including the participating Cree First Nations” (p. 2). The CBHSSJB Chairperson is elected by the Cree Nations’ Peoples and is the Cree representative on the CBHSSJB Board of Directors [20]. The CBHSSJB follows the OCAP (ownership, control, access and possession) principles and the Tri- Council Policy Statement (Ethical Conduct for Research Involving Humans) [23]. Prior to sending out manuscripts for journal peer review, as per the Research and Data Use Agreement [21], draft manuscripts had to undergo a review process with the CBHSSJB Data Analysis Review Committee, and “address comments and resolve any issues raised by reviewers” (p. 26).

Our study used data from five communities in the NA study – excluding data from the two pilot studies and two additional communities as the question about participants’ days spent on-the-land was not included in the questionnaire in these communities (Supplementary Figure S1). The age and sex data used in this study excluded those below the age of 15 because there were significant gaps in the NA study data for variables relevant to our study. Data for smoking status (essential for adjusting the inflammatory marker models) were also collected as a model-adjustment variable (Supplementary Figure S1). Based on this exclusion criteria, our study had *n* = 545 participants from five communities where data was collected from 2005 to 2009.

The health measures selected for analysis included: self-reported weekly activity (i.e. vigorous, moderate, and walking in hours per week (hours/week)); a proxy measure for traditional diet consumption (i.e. omega-3 polyunsaturated fatty acids in nanomoles per litre (nmol/L) [30, 119];); other lipids (i.e. total cholesterol, low-density lipoprotein (LDL) cholesterol, and high-density lipoprotein (HDL) cholesterol in nmol/L); three inflammatory markers (i.e. tumour necrosis factor-alpha (TNF-α) and interleukin-6 (IL-6) both in picograms per millilitre (pg/mL), and c-reactive protein (CRP) in milligrams per litre (mg/L)); and anthropometric measures (i.e. Body Mass Index (BMI) in kilograms per square metre (kg/m^2^) and waist circumference in centimetres (cm)). Data for cholesterol, LDL, HDL, TNF-α, and IL-6 were log-transformed. See Liberda et al. [62] for a detailed explanation of the biomarker laboratory analysis. Furthermore, blood concentrations of total mercury (nmol/L) were examined as a potential risk of being on-the-land, and the data were also log-transformed. A sensitivity analysis was also run for selenium’s potential alleviation effects on mercury impacts in the regression models.

The independent variable in our study was a binary variable for eligibility for participation in the ISP. Study participants were classified as eligible if 120 days or more were spent on-the-land performing traditional cultural activities during the previous year (i.e. in the last 365 days), and ineligible if 119 days or less were spent on-the-land over the previous year. We assumed eligibility for the ISP corresponded to the potential benefits of participating in the ISP, however, this independent variable is not a direct measure of actual participation. The inclusion criteria in our dataset corresponded to a participant with known age, sex, and smoking status, and at least one of the measures under investigation (Supplementary Figure S1); thus, sample sizes are different for some variables and do not total to the overall *n*. Table 1 shows the available sample sizes of participant data observed for each dependent variable categorized by the independent variable and sex.

**Table 1:** Descriptive statistics of participants based on the eligibility for participation in the Income Security Program (ISP)

	Income Security Program Eligibility							
	Ineligible to participate (≤ 119 days on-the-land)				Eligible to participate (≥ 120 days on-the-land)			
Variable	Male (***n***)	Female (***n***)	All (***n***)	Mean (SD)	Male (***n***)	Female (***n***)	All (***n***)	Mean (SD)
**Activity (hours/week)**^**a**^								
Vigorous	78	71	149	1.52 (1.80)	19	14	33	3.50 (2.43)
Moderate	101	124	225	2.03 (2.04)	24	22	46	3.71 (2.95)
Walking	99	113	212	1.87 (2.78)	23	14	37	1.70 (1.84)
**Anthropometry**								
BMI (kg/m^2^)	172	272	444	32.98 (7.37)	45	37	82	34.22 (8.80)
Waist circumference (cm)	173	272	445	107.99 (15.91)	45	37	82	112.20 (14.33)
**Traditional diet (nmol/L)**								
Omega-3	108	137	245	6.20 (1.51)	25	17	42	7.66 (1.61)
**Lipid (nmol/L)**								
Cholesterol	173	273	446	0.73 (0.07)	45	37	82	0.75 (0.07)
LDL	172	267	439	0.53 (0.10)	42	37	79	0.56 (0.09)
HDL	173	273	446	0.35 (0.06)	45	37	82	0.36 (0.05)
**Inflammatory Marker**^**b**^								
TNF-α (pg/mL)	108	137	245	2.94 (2.20)	25	18	43	2.77 (3.04)
IL-6 (pg/mL)	108	137	245	2.42 (1.99)	25	18	43	2.95 (1.79)
CRP (mg/L)	108	137	245	2.58 (3.17)	25	18	43	3.30 (2.59)
**Contaminant**^**b**^ **(nmol/L)**								
Blood [Hg]	173	273	446	15.57 (3.66)	45	37	82	63.94 (3.14)
Age mean (SD)	34.47 (15.74)	34.12 (34)	34.26 (15.49)		50.32 (18.03)	55.40 (15.04)	52.78 (16.76)	

*Note*:

^a^ categories are not mutually exclusive due to multiple responses to activity

^b^ geometric mean and standard deviation

*Key*: *BMI* body mass index, *Omega-3* omega-3 polyunsaturated fatty acids, *LDL* low density lipoprotein, *HDL* high density lipoprotein, *TNF-α* tumor necrosis factor-alpha, *IL-6* interleukin-6, *CRP* c-reactive protein, *[Hg]* mercury concentration

### Statistical analysis

Descriptive statistics of the variables are reported as arithmetic means and standard deviations (SD) – unless otherwise stated in Table 1.

Unadjusted-regression models with only one of the health measure variables and multivariable regression models using the same health measure variables, adjusted for age and sex, were carried out to assess the potential associations of ISP eligibility status of each participant with the health measures. Age and sex were included as covariates in the adjusted model as methylmercury is bioaccumulative [8] and men are more likely to be hunters and fishers compared to women [78]. Furthermore, smoking status was used to adjust the inflammatory marker models in addition to age and sex [61]. All models were checked for fit and strength, and the statistical analyses for our study were carried out in R version 3.6.2 [91]. Statistical significance was set at *p* <  0.05.

## Results

### Descriptive results

The overall mean age of Cree Peoples eligible for participation in the ISP program is approximately 53 years old compared to 34 years old for ineligible participants, and those who are eligible to participate in the ISP are more frequently males. Table 1 displays the means and standard deviations from the health measure variables categorized by the ISP eligibility variable. The table also presents the differences for each variable between eligible and ineligible participants for the ISP.

### Regression modelling

Unadjusted and adjusted model beta coefficients from the regression models between the health measure variables and eligibility to participate in the ISP are presented in Table 2. From the adjusted models of eligible ISP participants, the results were significantly higher for vigorous (β = 1.623 (95% CI: 0.814–2.431), *p* <  0.001) and moderate activities (β =1.556 (95% CI: 0.780–2.333), *p* <  0.001), but not walking (Table 2). Cree Peoples eligible for participation in the ISP had a significantly higher association with omega-3 polyunsaturated fatty acids (β = 0.534 (95% CI: 0.188–0.879), *p* = 0.003) compared to those Cree participants ineligible for the ISP (Table 2). Cree Peoples who were eligible for participation in the ISP were not statistically different from ineligible participants for the ISP for total cholesterol, LDL, and HDL. As well, no significant results were found for inflammatory markers TNF-α, IL-6, and CRP, or anthropometrics (i.e. BMI or waist circumference). For Cree Peoples who were eligible to participate in the ISP, their blood concentration of total mercury was not significantly higher than those ineligible to participate in the ISP, after adjusting for age and sex. The sensitivity analysis with selenium was not significant and did not affect the regression model result for mercury impacts (Supplementary Table S1).

**Table 2:** Linear regression model results assessing the association of eligibility to participate in the Income Security Program (ISP) with specific health measures

Health measure	Unadjusted beta-coefficient		Adjusted beta-coefficient	
	β_1_ (95% CI)	*p*-value	β_1_ (95% CI)	*p*-value
**Activity (hours/week)**				
Vigorous	1.977 (1.252–2.703)	**< 0.001**	1.623 (0.814–2.431)	**< 0.001**
Moderate	1.675 (0.973–2.378)	**< 0.001**	1.556 (0.780–2.333)	**< 0.001**
Walking	− 0.171 (− 1.101–0.760)	0.719	−0.068 (− 1.079–0.943)	0.895
**Anthropometry**				
BMI (kg/m^2^)	1.236 (− 0.556–3.028)	0.177	0.812 (− 1.046–2.670)	0.392
Waist (cm)	4.210 (0.517–7.902)	**0.026**	0.284 (− 3.572–4.139)	0.885
**Traditional diet (nmol/L)**				
Omega-3	1.503 (1.052–1.954)	**< 0.001**	0.534 (0.188–0.879)	**0.003**
**Lipid**^**a**^ **(nmol/L)**				
Cholesterol	0.013 (0.003–0.023)	**0.014**	0.001 (− 0.010–0.011)	0.880
LDL^a^	0.027 (0.004–0.049)	**0.022**	−0.002 (− 0.026–0.021)	0.840
HDL^a^	0.002 (− 0.011–0.016)	0.730	0.001 (− 0.013–0.016)	0.874
**Inflammatory Marker**				
TNF-α (pg/mL)^a^	−0.027 (− 0.146–0.092)	0.658	−0.024 (− 0.151–0.103)	0.714
IL-6 (pg/mL)^a^	0.086 (− 0.009–0.180)	0.078	0.093 (− 0.005–0.191)	0.063
CRP (mg/L)	0.109 (− 2.491–2.708)	0.935	0.444 (− 2.357–3.245)	0.756
**Contaminant (nmol/L)**				
Blood [Hg]^a^	0.297 (0.173–0.422)	**< 0.001**	− 0.052 (− 0.171–0.066)	0.385

*Note*:

Bold indicates statistical significance at *p* < 0.05

Unadjusted model: health measure dependent variable only

Adjusted model: unadjusted model + age, sex, smoking (smoking added to inflammatory marker models only)

^a^ log-transformed

*Key*: *BMI* body mass index, *CI* confidence intervals, *Omega-3* omega-3 polyunsaturated fatty acids, *LDL* low density lipoprotein, *HDL* high density lipoprotein, *TNF-α* tumor necrosis factor-alpha, *IL-6* interleukin-6, *CRP* c-reactive protein, *[Hg]* mercury concentration

## Discussion

Traditional on-the-land cultural activities have been recognized in several studies as being vital to health and well-being for those who participated [42, 63, 82, 96, 126], but quantitative studies including health measures are lacking. Our findings show that Cree Peoples who are eligible for participation in the ISP reported a higher level of physical activity (i.e. vigorous and moderate activities) and had higher levels of omega-3 polyunsaturated fatty acids (an indicator of traditional food consumption), and similar blood concentrations of mercury compared to those ineligible for participation following model adjustment. Interestingly, in non-Indigenous populations the importance of greenspace [65, 68, 70, 120, 128] and water [28]; Horizon 2020 “Blue Health” project; [125] has been recognized. Additionally, the health and wellbeing of Indigenous Peoples living in an urban environment is an emerging field [43].

### Activity levels and anthropometrics

Programs that include physical activities based in Indigenous communities have yielded positive results, such as, decreased obesity, improved type 2 diabetes status and a reduction of cardiovascular diseases [4, 16, 34, 52, 53, 59, 102]. Our study demonstrates that Cree Peoples, who were eligible for participation in the ISP, had higher levels of vigorous and moderate physical activity over those Cree Peoples who were ineligible for participation. These results are similar to those of Robinson et al. [97], who reported in a prospective cohort study with the James Bay Cree Peoples that participants were more active when on-the-land, and more active than a community-based control group of Cree Peoples in *Eeyou Istchee*. An increase in moderate-to-vigorous physical activity may increase life longevity, and be cardioprotective [60, 94, 108]. As a result, there has been a trend to include traditional cultural activities in programs for Indigenous communities, because the potential benefits of traditional cultural activities go beyond physical health (e.g. mental health and well-being) [26, 47, 103, 107, 123].

Interestingly, our study did not demonstrate improvement in BMI or waist circumference of participants eligible for the ISP even though previous studies demonstrated some improvement in various anthropometric measures with increased physical activity [14, 38, 99]. In the Robinson et al. [97] study, the authors reported that the mean BMI for the on-the-land group was significantly lower than that of the community-based control group, but within the on-the-land group post-measures for weight were not significant. Nevertheless, participation in the ISP program promotes both vigorous and moderate activities in land-based traditional cultural activities – while supplementing income – an additional benefit. Increased physical activity as a result of on-the-land programs, like the ISP, are also successful because they are rooted in the local culture and provide a meaningful way of engaging in traditional cultural activities [11, 86, 111].

### Traditional diet (omega-3 polyunsaturated fatty acids)

Diet is a unique and essential feature of the traditional cultural lifestyle for the Cree Peoples of northern Quebec, particularly the dietary composition of fats and fat components, which are considerably different from a western diet [10, 27, 29, 50, 51]. It has long been established that Indigenous community members in northern regions who consume traditional foods high in omega-3 polyunsaturated fatty acids have a significantly reduced risk of vascular diseases [6, 7, 29, 32]. Studies have demonstrated that when Indigenous community members revert to a traditional cultural lifestyle – even for intermittent periods – their risk of type 2 diabetes and cardiometabolic problems decreases significantly because animals available to the hunter and/or trapper are low in undesirable fat with higher levels of beneficial fatty acids [58, 69, 78, 79, 83, 88]. In our study, we determined that those eligible for participation in the ISP have significantly higher omega-3 polyunsaturated fatty acid levels compared to those ineligible for the ISP, which likely indicates that eligible participants are eating more fish, as fish is highest in omega-3 polyunsaturated fatty acids [88]. Omega-3 polyunsaturated fatty acids are known to have myriad health benefits, namely reducing cardiovascular diseases, cholesterol and LDL, and having anti-inflammatory and neurologically protective effects [29, 31, 64, 100, 121]. Furthermore, increased levels can even be associated with improved mental well-being [57]. Although omega-3 polyunsaturated fatty acids are found in wild game meats [104], land-based animals and birds native to this region tend to have higher levels of omega-6 polyunsaturated fatty acids [89, 95], which suggests that the increased levels of omega-3 polyunsaturated fatty acids are from the consumption of fish for those eligible to participate in the ISP. This finding is important because eligible participants are engaging in traditional cultural activities and benefit from an increase in healthy dietary fat.

### Lipids (Total cholesterol, LDL cholesterol, HDL cholesterol)

Increased cholesterol and LDL levels are associated with a higher risk of vascular events (e.g. myocardial infarction, stroke, or occlusive vascular diseases). These vascular events are typically a result of consuming foods of low nutritional value related to a westernized diet, or cooking methods that promote the use of unhealthy fats [5, 15, 44, 67, 110]. Lifestyle and genetics may also increase cholesterol and LDL levels [41, 55, 66]. Given the increased blood level of omega-3 polyunsaturated fatty acids for participants eligible for the ISP in the present study – and the higher levels of vigorous and moderate physical activity − we expected to see a decrease in cholesterol and LDL for eligible participants of the ISP compared to those ineligible. However, our results did not show this, as unadjusted and adjusted models were non-significant. Perhaps the cross-sectional design of the study contributed to this non-association.

Increased HDL levels are related to a traditional cultural diet, as Young [129] demonstrated in a culturally similar Indigenous population; since eligible participants for the ISP are more likely to consume traditional foods containing beneficial dietary fatty acids and other nutritive elements, it would be thought that those eligible for the ISP would have significantly higher HDL levels. Higher levels of HDL are associated with lower levels of cholesterol and LDL, and therefore, there is an assumed lower risk of occlusive vascular events and other cardiovascular health problems in people with optimal HDL [40, 67, 127]. However, our study results demonstrated no evidence of higher HDL levels for Cree Peoples eligible for participation in the ISP compared to those ineligible for participation in ISP. Again, we believe this non-significant result may be a consequence of the cross-sectional nature of the data collection.

### Inflammatory markers

Inflammation in the human body can be examined through markers in the blood that indicate there is either acute or chronic inflammation and may indicate specific illness or disease, or an exposure-response to a xenobiotic [37, 124]. The cytokine TNF-α is a pro-inflammatory marker, and is involved in acute or chronic inflammation in the human body [90, 122]. IL-6 is an inflammatory cytokine that can act as a defence mechanism, and be an indicator of acute or chronic inflammation [35]. Furthermore, CRP, an acute-phase protein synthesized in hepatocytes mainly in response to IL-6, is a predominant marker of acute inflammation rather than chronic inflammation [85, 105]. All of these inflammatory markers may increase due to internal or external stimulus and are associated to many different exposures, risk factors, or illnesses of the human body (e.g. obesity, cancer, diabetes) [9, 87, 118].

In our study, the results for inflammatory markers were not significant between groups. Perhaps, a lack of differences of inflammatory markers between groups in our study could be related to physical activity intensity, which is known to cause acute inflammation in the human body up to 24 h following activity – especially if the activity is moderate or vigorous [33, 54, 84, 101]. We found that Cree Peoples eligible to participate in the ISP had increased levels of moderate and vigorous activity, so there could be an association to increased inflammation as a result. However, we are unable to fully confirm this latter association as we do not have information regarding the exact timeframe from when the participant was last on the land, and when they had their blood sample drawn for analyses of inflammatory markers.

When investigating the variables used to adjust the inflammatory marker regression models, we found that smoking was the only significant variable associated with inflammation, which suggests that those who smoke are more likely to have increased levels of TNF-α, IL-6, and CRP [2, 56, 80]. Furthermore, those who are on-the-land 120 days or more per year, and therefore eligible for participation in the ISP, may have increased exposure to wood smoke from burning wood for heating and cooking, which is known to increase inflammatory marker levels upon exposure [12, 73, 109].

### Mercury

Exposure to mercury from the environment, methylmercury from fish consumption mainly, has the potential for neurotoxic effects in humans [17–19]. Previous studies of methylmercury in the study region found that certain species of fish and community location in the study region [71, 72] influenced the risk of being exposed to higher levels of methylmercury. The consumption of medium and large-sized predatory fish, and participants who reside in communities nearby to high-intensity industrial land use on traditional homelands were at the greatest possibility for exposure to methylmercury according to those studies. Encouragingly, our study results indicate that Cree Peoples who are eligible to participate in the ISP, do not have increased blood concentrations of mercury compared to those who are ineligible to participate in the ISP. This finding is noteworthy because we assumed that those who spend more time on the land may have been eating more fish; thus, participants would be expected to have a higher body burden of mercury even after age-adjustment, but this was not the case. Nonetheless, we encourage community members to follow fish consumption recommendations as advised by the CBHSSJB [22] to minimize mercury exposure.

### Limitations

The use of data from a cross-sectional study design was a limitation, and the type of data collected limited our ability to adjust models beyond age, sex and smoking status (for inflammatory markers only). Some variables of interest had missing data; thus, sample sizes for some variables were limited. The limitations above may have also influenced the results of the linear models. Additionally, this study addresses a knowledge gap, but only from a non-Indigenous health measures perspective. Ideally, Indigenous measures of health and well-being should have been collected (e.g. the vertical and horizontal transmission of Indigenous knowledge, the re-establishing and strengthening of social networks, and the general feeling of wellness out on-the-land [113]). Nonetheless, the relatively large overall sample sizes of this study for an Indigenous nation, and the evaluation of the longest continuously running on-the-land intervention globally is unique.

## Conclusions

We present novel cross-sectional results of health measures for northern Quebec First Nations Cree Peoples who are eligible to participate in the on-the-land ISP. The health measures results were promising for those eligible to participate in the ISP. Specifically, higher levels of vigorous and moderate physical activity and higher levels of omega-3 polyunsaturated fatty acids for individuals who were eligible to participate were found – while no differences in mercury blood concentrations between Cree Peoples who were eligible compared to those ineligible to participate in the ISP were found. Therefore, the results of our study encourage on-the-land traditional cultural activities for Cree Peoples – and other Indigenous peoples – and support on-the-land mitigation and/or intervention activities to ensure their health and well-being when on-the-land.

## Supplementary Information

**Additional file 1.**

## Data Availability

All data that support the findings of this study are retained with the Cree Board of Health and Social Services of James Bay. The data are not publicly available or available upon request because they contain participants’ confidential health data.

## Abbreviations

BMI: Body mass index
CI: Confidence interval
CRP: C-reactive protein
HDL: High-density lipoprotein
IL-6: Interleukin-6
ISP: Income Security Program
JBNQA: James Bay and Northern Quebec Agreement
LDL: Low-density lipoprotein
NA Study: *Nituuchischaayihtitaau Aschii* Multi-Community Environment-and-Health Study in *Eeyou Istchee*
TNF-α: Tumour necrosis factor alpha
