# Supplementary material for: Health measures of Eeyouch (Cree) who are eligible to participate in the on-the-land Income Security Program in Eeyou Istchee (northern Quebec, Canada)
Source: BMC Public Health. 2021 Mar 31;21:628. doi: 10.1186/s12889-021-10654-7 (PMC8011104; doi:10.1186/s12889-021-10654-7)
Supplement: Supplementary file 1 — Additional file 1. [file 12889_2021_10654_MOESM1_ESM.docx]

**Supplementary material**

**Supplementary Table S1. Linear regression model results assessing the joint association of selenium blood concentration and eligibility to participate in the Income Security Program (ISP) with the mercury blood concentration health measure.**

| **Health measure** | **Unadjusted beta-coefficient** | | **Adjusted beta-coefficient** | |
| --- | --- | --- | --- | --- |
|  | β_1_ (95% CI) | *p*-value | β_1_ (95% CI) | *p*-value |
| **Contaminant (nmol/L)** |  |  |  |  |
| Blood [Hg]^a^ | -0.372 (-0.309 – 1.617) | 0.678 | -0.451 (-1.866 – 0.965) | 0.533 |

*Note:*

Bold indicates statistical significance at *p* < 0.05.

Unadjusted model: health measure dependent variable only.

Adjusted model: unadjusted model + age, sex.

a: log-transformed.

*Key*: [Hg]: mercury concentration.

**Supplemental Figure S1. Flowchart of participants included in the study analysis, data from the cross-sectional *Nituuchischaayihtitaau Aschii* Multi-Community Environment-and-Health Study (2005 – 2009) in the *Eeyou Istchee* territory northern Quebec, Canada.**

Multi-Community Environment-and-Health Study (*N* = 1405)

Participants 15 years of age and older with corresponding age and sex (*n* = 971)

Participants less than 15 years of age (*n* = 434)

Participants without known days on-the-land data (*n* = 299)

Adults with a complete study profile (*n* = 545)

Females (*n* = 321; 59%)

Males (*n* = 224; 41%)

Adults with known smoking status (*n* = 844)

Participants without known smoking status data* (*n* = 127)

* crucial for adjusting inflammatory marker regression models
